# Letter from Paris

**Published:** 1857-12

**Authors:** 


[From the New Orleans Medical News and Hospital Gazette.]

### *Letter from Paris.*

Paris, August 30th, 1857.

Messrs. Editors: An humble pilgrim at the shrine of science, I bring you a few gleanings gathered among the "on dits" in medical circles. But this is a period of repose in the Schools; the annual harvest is gathered, and the master reapers—the Velpeaus, the Nelatons, the Trousseaus—have left the field of their labors to prepare for the coming campaign. You will at once understand that, the rich mine of medical instruction being suspended, we must turn from the hospitals and the amphitheatres to other sources of medical news. But though we must thus leave out of the question Practice and Surgery, we can turn to other sources and find that many a diligent laborer yet remains, still delving in the fields of knowledge, as if impatient at Time's unceasing flight, or eager, before their journey of life is o'er, to raise a corner of that impenetrable curtain which conceals from our sight as well as the secrets of the future many of the mysteries of our organization.

But as the morrow of to-day will soon have passed, and enrolled itself in the archives of historical lore, with its tale of weal or woe henceforth indelibly inscribed, we must be content to seek among these teachings of the past the assurance that progress and perfection should be the motto of man. Among those subjects which have more immediate relation to man himself, certainly none deserve a more important place, in its immediate bearings upon ethnological science, than the labors of the Physiologist; and here I would embrace not only those considerations which are purely relative to the laws of organic functions, but all those questions of Hygiene, Embryology, Histology, and intellectual capacities, all of which have so important a bearing upon the origin of man, his development, his capacities, his progress, and that scarce attempted question, his end and object, as a part of creation. It cannot be denied that in this programme of physiological inquiry, there are many points which are wrapped in primitive darkness. Let us, however, remember, that ages are nothing in the progress of truth, and that just as social and political reforms are the slow results of ages of abuse and tyranny, so the best established axioms in medicine have been the most contested, and the tardy growth in experience and observation. The nineteenth century may justly claim pre-eminence over others for the importance of its discoveries in physiology and medicine. It is only of late years that the experimental method has been introduced into physiology, by the illustrious Magendie ; and yet it has already revolutionized the dogmas of the schools, either by upsetting old theories, or confirming them by the rigid laws of demonstration, or introducing new ideas of which not even the germ had pre-existed.

It would be a most interesting though arduous task to assign to each nation the share which it deserves in the progress of physiological knowledge. Thus, starting from Haller, the greatest representative of the modern school, we might pass in review the influence of the German schools, the propagation of their labors in England, and America ; nor should we forget the Fontanas, the Matteucis, and other distinguished Italian physiologists. But we must undoubtedly assign the palm to that far famed French school, which, starting with Bichat, continues its brilliant galaxy with the names of Legallois, Richerand, Magendie, Berard, Beclard, Robin and others, until we arrive at the present time, and find the sceptre of physiology in the hands of Claude Bernard.

It were no easy task to bear the mantle which had been worn by so many distinguished predecessors, yet all Europe has acquiesced in that apostolic succession, and few but the envious or ignorant would gainsay the choice. Bernard is essentially the experimental physiologist of the day, and he has attained his present proud eminence by 20 years of indefatigable labor and research. Before Bernard, before Magendie himself, Legallois had led the way by experiments upon living animals, though we might turn further back to the remarkable era when Harvey demonstrated before a monarch the circulation of the blood on a living fawn. There can be no doubt, also, that Haller discovered by actual vivisections the the doctrine of irritability and excitability, which he first propagated to the scientific world ; still these were but isolated facts, and no regular method or system had as yet been adopted or pursued. Bichat, the immortal author of the Researches on Life and Death, was more of a pathological anatomist than a physiologist, and it is probable that he built up his great work on general anatomy, of which he was the founder, by observations on the phenomena of disease. Endowed with a gigantic intellect, Bichat unfortunately allowed his fondness for system and theory to allure him from the narrow road of observation and reality. He accounted for all the phenomena of living bodies by supposing that each tissue was endowed with certain vital properties, from the mutual play and coordination of which resulted life, considered as a whole; whereas, physical and chemical actions were peculiar and inherent to the inorganic world. It is scarcely necessary now-a-days to refute such a proposition, when we remember that digestion is a purely chemical phenomenon. But Bichat died at the early age of 32, and we may easily understand that the immense structure whose foundations he thus laid, must have been wanting in harmony and strength. Legallois, the cotemporary of Bichat, survived him a few years, and contributed many important additions to our science, yet he is scarcely known at the presen. day, though his "Experiments on the Principle of Life," is a most ret markable production, and a translation appeared in Philadelphia, where he was chosen corresponding member of the Medical Association in that city.

Bichat died in 1820; in 1808 Magendie appeared upon the stage, and during forty years the torch, with which he explored so many obscure questions, never wavered in his hands, or wandered from the strict path of observation and experiment. Systematically opposed to the method of generalization and dogmatic theories which had been so much the fashion before his time, Magendie adopted the strict interpretation of nature, and only admitted as a fact that which he had corroborated by observation; so that he never departed by analogy or hypothetical reasoning, from the stern reality of experimental deduction. His devotion to this method, which he admitted as the sole criterian of truth, may sometimes have been pushed too far, yet it could scarcely mislead him long, and he has often been accused of inconsistency when he had barely related experiments which, as so often happens in physiology, were apparently in opposition with each other. Such, for instance, were his remarkable labors on the functions of the anterior and posterior roots of the spinal nerves. He saw that sometimes the anterior roots were endowed with sensibility; at other times they appeared insensible. In later times his disciple and successor, Bernard, was the first to show conclusively, that this peculiarity depends upon the so-called reflex function of the nervous system, and that it varies according to the point where the section of the motor nerve happens to be made. Thus it was that Magendie invariably stated the facts as he saw them, without ever attempting to distort them in order that they might serve some preconceived opinion or theory.

When Fred Tiedmann, the great German physiologist, visited Paris several years ago, he called upon Magendie, who was then occupied with his researches upon the cerebro-spinal fluid. The French Professor explained to his guest that the cerebro spinal fluid was interposed between the nervous centres and the arachnoid; that it is continuous along the spinal cord, where it subserves the important function of protecting those delicate textures from any sudden or violent shock. But the German philosopher at once objected. "It is impossible," said he, "that this can be so, for has not Bichat established the law that all liquids must be contained within a serous cavity? Now, the arachnoid being a shut sac, this liquid evidently must be within the membranes." I do not care about Bichat's law, answered Magendie, but I will certainly show you that the cerebro-spinal fluid is beneath the reflected portion of the arachnoid; and this is accordingly the fact now universally admitted. Magendie believed that we were removed from the time when any general dogmatic treatise could be written on physiology, and he considered that the science was far from being firmly settled on its foundation; and that generalization should only be followed when a sufficient number of uncontested facts have been accumulated. The vanity of men, he said, often leads them to compare themselves, each in his own sphere, to some great name or object to which they fancy themselves either approaching or similar. Some have compared themselves to Archimedes, or to Michael Angelo, or a Newton, or a Galileo. Louis XIV compared himself to the sun; but for my part I am more humble in my simile. I fancy myself like a ragman, for with a hod on my back, and stick in hand, I go through the by-ways of science, picking up all the detached facts I may find on the road. Such was Magendie, and such was the tendency and object of his labors. His severe and unbending devotion to experimental truth was necessary at the time in which he lived, and he is justly entitled to the credit of having diffused throughout Europe his methods of vivisection, to which we owe those rapid strides that of late years have signalized the progress of physiology. If we may believe that a pure and etherial essence survives the dissolution of an earthly mould, we can fancy that the spirit of this great man yet hovers around that laboratory of the College of France, where for forty years he pursued his researches in the properties of organized beings. But the genius which guided him through his long ministry has certainly survived him, and the discoveries of Bernard have even eclipsed those of his master and predecessor. From his present exalted position, though he might safely rest on the laurels he has secured, with true devotion to the cause of science, he only considers the achievements of the past as motives for future exertion; and he is now as unremitting in his labors as if he were at the threshold of the temple of which he is now the grand high-priest. Nor does he sit in his chair with unalloyed peace ; more than one parasitic insect has attempted to buzz himself into notoriety by attacking the writings of Bernard. Their object is attained if they attracted a passing notice from the press of the day, and their highest ambition is to secure a report on their hybrid productions from the Academy of Sciences. But these pigmies soon sink into that oblivion from which they strive to raise themselves by their noisy though empty clamors. But every true lover of the science will learn with regret that a great name has also lent itself to a series of attacks as violent as they were unfounded. I mean Pierre Berard, Alas ! the Berard of to-day is not the same as the author of the Lectures on Physiology—the same in person, but not in mind. Last year he had a severe attack of apoplexy, and I fear some softening of the brain consequent thereon, now incapacitates him for that impartial and just appreciation of men and their works, which was so eminently characteristic of the learned professor of the Faculty. He has undertaken a series of experiments to disprove Bernard's theory on the function of the pancreatic juice, and lately published the result of his labors in the shape of a violent philippic against the Professor of the College of France. Berard associated with him in his unenviable labors, a certain subaltern veterinary of Alfort, who has undertaken the anatomical part of the experiments; for Bernard, it is well known, has never performed a vivisection in his life, and his book was written from the materials accumulated by others. In this case, his choice of a Moderator has been most unfortunate, for I have heard two of the Professors of Alfort, Messrs. Chauveau and Boulay, declare that they have not the least confidence in any experiment of their subaltern, Colin ; and it is well known that he will distort any fact to suit his purpose. In the present case, he pretended that a ligature of the pancreatic duct does not prevent the digestion and absorption of fatty substances. This assertion will, no doubt, appear monstrous, yet the hardihood of its author is only equalled by the falsity of his conclusions ; nor would it attract more notice or belief than the assertion of some pretended astronomer, who revived a short time ago, the old exploded idea that the sun revolves around the earth, if it were not that a great though fallen mind has associated his name to these ill-shapen and monstrous productions.

At the same time that the discoveries of Bernard have placed the French Schools of Physjology above all others, so that we can well assert that the nineteenth century has paid its debt to the ever accumulating fund of human science, and realized many important steps in the law of progress, we can even look beyond the present time and discover the promises of the future. It would seem as if the indefatigable Professor of the College of France where determined to leave no part of physiology unexplored, for, besides the discoveries which have already made him famous, viz: those on digestion, the pancreatic juice, the saliva, the functions of the liver, the secretion of sugar in that organ, and many others besides, he is now preparing two volumes on the Physiology of the Nervous System, which are expected to be published sometime in November, and these will be no doubt the most complete monographs ever published on these obscure and complicated questions. But when I speak of the future, I do not mean even these, for they are a continuation of his admirable labors, and those who have had an opportunity to follow his lectures or attend his private experiments are already acquainted with many of his conclusions on the functions of the nerves. There is another department of physiology, which is destined at no distant day to shed new light on the organization and properties of living beings—I mean microscopical science. The microscope, as an instrument, has long been known, and every one is aware that De Graaf, Lewenhoeck, Malpighi, and many others successfully applied this instrument to the study of organized bodies; but it is only of late years that histology, or the application of the microscope to the study of the different textures of the human body, has really grown into a science. But. as a science, it has now acquired an independent growth, requiring for its mastery the most laborious and untiring industry. It is impossible at this day to give a correct appreciation of the value of microscopical investigation, for its deductions are yet so far removed from the standard of ultimate perfection, that many, even fundamental questions, are as yet mooted points with many writers, although, to the practiced and enlightened microscopist, the applications of this instrument are as unerring as those of the eye itself in those grosser structures where the latter needs no such assistance. One of the causes of popular objection to the microscope, is the belief, common to all beginners, that any of the tissues of the body can be at once recognized by simply putting a specimen supposed to contain this or that element under the objective of no matter what magnifying power. Now the truth of it is, that it is exceedingly difficult, in the first place, so to separate and dissociate many of the different elements which enter into the composition of a given structure, that it may be recognized by the unpracticed eye. Take, for instance, a piece of muscular tissue; perhaps you will expect to recognize at once the ultimate fibril of animal life; you will desire to see the difference between the voluntary or striated, and the involuntary or smooth muscle. You read in almost any text book that such is the fundamental element of these two varieties of muscular tissue, and that its contractility depends upon that very element, at the same time that another tissue is so disposed as to restore the fibril to its former position; you will see that your author has employed the pencil of some cunning artist to depict for you with all the delicacies of light and shade, the transverse and longitudinal striæ; well, perchance you have secured one of these instruments, yclept a microscope, and scalpel in hand, you are prepared to dive into all the mysteries of organization and life, you will verify if Carpenter, Queckett and Hassel have correctly delineated these structures. You secure a splendid piece of muscle, and you put a specimen under your objective. Alas! cruel deception, your preparation is too thick; the light does not penetrate, and you see nothing but a dark and confused mass; after many efforts you have succeeded in dilacerating your preparation so that little if anything apparently is left on your object-glass, and again you examine attentively this elaborate preparation, and even then you may be doomed to disappointment until, in utter despair, you come to the conclusion that the microscope is a useless instrument, and micrographers very little better than humbugs. The conclusion is natural enough; and yet, it is false as it is unjust; it is false because what you have read was the result of a preparation by a master hand, who selected from scores of preparations, what he considered the type of the element he described; it is unjust, because you have no right to expect that in an hour or a day you can acquire that dexterity of manipulation which is the slow result of practice and experience. Have you forgotten the time when your first subject was on the table before you, and armed with your scalpel, you were about to take your first lesson in practical anatomy? for the first time you were face to face with death. Your overcame your scruples with no matter what unsteady hand, and you commenced imitating either those around you, or the skillful dissection of your demonstrator; you saw him exposing before you with rapid and unerring skill the fibres of muscle after muscle, but when you resumed the task how different your production from the artistic execution you had just witnessed. Did you throw away your knife in disgust, or fancy your fingers were differently made from those around you? No! you persevered until you acquired sufficient dexterity for all practical purposes. Now, there is no question but that microscopal dissection is far more difficult than the other, and a very little reflection will at once convince us of the nature of the difficulty in hand. We are applying an instrument which magnifies several hundred times, perhaps, to investigate the very secret of life, the intimate nature of that fabric which is the master piece of creation, and we can reasonably expect that we shall at once lift the veil, and surprise the essence of that being without due preparation and initiation? The learning which is so easily acquired, is not worth having. No, in that investigation, no matter what texture, even the simplest, we can not expect that its elements will be presented to us perfectly isolated and distinct. Far from it, they are so blended and interwoven, that it requires much patience, and often renewed efforts to separate them one from the other, or to recognize them when they have been at last obtained with satisfactory distinctness. Let us return, for instance, to our piece of muscle, and see of what it is made up; in it are many different elements besides the fundamental or constituent element, viz: 1. The myolemma or investing sheath of each fibril. 2. Elastic fibres. 3. Cellular tissue. 4. Cells containing fatty matter. 5. Capillary vessels of three different kinds. 6. Arteries and vein. 7. Nerves. Now, can we reasonably expect that we shall at once distinguish all these, and recognize them even when the preparation is made by a master hand; by no means; for the eye must be educated to interpret that which is presented to it in the field of the microscope; it must be taught to recognize the elements when separated, and then we can better appreciate the aws of texture, that is to say the reciprocal arrangements of these elements, one with the other. Just as the ear of the practiced musician, or the eye of the painter, distinguishes nice shades of color, delicate intonations of sound, which escape the uneducated, so will the eye of the microscopist distinguish the fundamental element of a healthy tissue, or recognize the abnormal development of some other product in a diseased condition. But, however difficult the study of microscopal anatomy, it is replete with interest of the most profound and satisfactory kind, and the more we advance in its study, the more we rise in admiration of that great First Cause whose wisdom is incomprehensible, whose greatness incommensurable.

Carpenter, in his admirable book on the Microscope, has paid a just tribute to this instrument as a means of scientific research, and I cannot do better than quote his eloquent lines upon the subject. "As an optical instrument, the microscope is now at least as perfect as the telescope: for the six feet parabolic speculum of Lord Ross's gigantic instrument is not more completely adapted to the astronomical survey of the heavenly bodies, than the achromatic combination of lenses, so minute that they can scarcely be themselves discerned by the unaided eye, is to the scrutiny of the physiologist into the mysteries of life and organization.

Nor are the revelations of the one less surprising to those who find their greatest charm in novelty, or less interesting to those who apply themselves to the study of their scientific bearings, than are those of the other. The universe which the microscope brings under our ken, seems as unbounded in its limit as that whose remotest depths the telescope still vainly attempts to fathom. Wonders as great are disclosed in a speck of whose minuteness the mind can scarcely form any definite conception, as in the most mysterious of these nebulæ whose incalculable distance baffles our hopes of attaining a more intimate knowledge of their constitution. And the general doctrines to which the labors of microscopists are manifestly tending in regard to the laws of organization, and the nature of vital action, seems fully deserving to take rank in comprehensiveness and importance with the highest principles yet attained in physical or chemical science." Such then is the nature of microscopal investigation, and such the promises it holds for the future, and at no distant date we may expect to hail the fulfilment of these promises.

Much as Koelliker, Lebert, Mand, and many other distinguished names, have added to this important branch, there was still much obscurity, confusion, and contradiction in their writings, nor was there sufficient method in their investigations; and, above all things, they had not properly defined the relation between general anatomy and physiology. It is not often that one mind can be so far successful as to originate and perfect one branch or department of human knowledge. True, Læennec, the discover of auscultation, had so far completed his immortal work, that he left very little to subsequent investigators. But surely he who introduced method where chaos previously had ruled supreme, he who classified the morphological elements of the body, and distinguished the fundamental from the accessory, he who was the first to show the origin, the development, and decay of every tissue, its nutrition and its growth; such a man is entitled to no ordinary credit, and I need scarcely say, that wherever medicine is studied as a science, and physiology considered its true ground work, there the name of Charles Robin is honored and respected. And yet the time has not arrived when full justice can be awarded to the genius and talent of this extraordinary man. Science has never beheld a more ardent devotee, Truth a more unflinching champion. Combine these qualities with intellectual qualities of the highest order, and you can readily conceive what such a man may accomplish. Though he has been giving public lectures on General Anatomy for many years, he has not published any dogmatic treatise on the subject. He has been, however, accumulating materials with assiduous care during all this time, and the work, now in the hands of the printer, will probably be complete sometime next fall. Others, more ambitious of publicity than scrupulous about scientific accuracy, have sent out to the world productions which, though remarkable in themselves, are nevertheless, wanting in that mature and harmonious co-ordination which is the slow result of time and experience. His forthcoming work on Histology is the fruit of the most careful and laborious investigation of every tissue, normal and pathological, entering into the composition of man; and there can be no doubt that the profession will hail its advent with joy, as a sure and infallible guide in these delicate questions.

Mr. Robin has already published several other works, which have secured for him a most exalted reputation, at home and abroad. A late number of the American Journal of Medical Sciences, contained an able review of his Treatise on Chemical Anatomy, a work remarkable for its elaborate, yet lucid exposition of the Physiology and Chemistry of the tissues; for, as he has himself said, he undertook this work as an introduction to his General Anatomy.

The object, said he, of my Treatise on Chemical Anatomy, is to enable the anatomist and the physician to know exactly the intimate molecular composition of any organized body, in either of its three forms, whether solid, semi-liquid, or liquid. So that the object of the work is, to examine the organic matter of all the particles or elementary principles which, by their molecular union, compose these different substances. He does not attempt the study of organized matter itself, but of the elements which enter into its composition. He therefore proposes to reduce organized matter into its constituent parts, and to examine the properties which are peculiar to these elements taken separately, or irrespective of their position in the animal economy. He defines the elementary particles of organized substances to be those which are the result of true anatomical analysis in contradistinction to chemical analysis. These particles must be studied separately and alone by mechanical separation, but not by destructive agencies. This statement at once explains the aim and object of the work as distinguished from other treatises, and it is certainly one of the most original productions on this important subject. Mr. Robin has also published an excellent manual on the use of the Microscope, explaining at the same time its optical principles and practical uses, being a most excellent introduction to Microscopic Science. Many of his lesser works have been published in the Memoirs of the Society of Biology; and his edition of Nysten's Dictionary of Medicine, which he entirely re-wrote and re-modeled, is a monument of his vast learning and untiring industry. This book, though professing to be merely a Dictionary of Medical Terms, is also an excellent epitome of almost every head which is there treated; and the different articles on Histology are considered the best authority on this important subject. The work was so far successful that in the course of two years an enormous edition has been almost entirely disposed of, his fortunate publisher realizing some thirty thousand francs from the operation. Sic von, non vobis, etc.

During all the summer, Dr. Robin has been employed npon these two works, viz: a new edition of the Dictionary of Medicine, and his Treatise of General Anatomy; but he also delivered an excellent course of lectures on the latter subject, and we hope, at no distant day, to submit our notes thereon to the readers of the Gazette. A. P.

